# Correlation between snoring sounds and obstructive sleep apnea in adults: a meta-regression analysis

**DOI:** 10.5935/1984-0063.20220068

**Published:** 2022

**Authors:** Jui-Kun Chiang, Yen-Chang Lin, Chih-Ming Lu, Yee-Hsin Kao

**Affiliations:** 1 Dalin Tzu Chi Hospital, Family Medicine - Chiayi - Taiwan; 2 Nature Dental Clinic, Dental department - Puli - Taiwan; 3 Dalin Tzu Chi Hospital, Department of Urology - Chiayi - Taiwan; 4 Tainan Municipal Hospital (Managed by Show Chwan Medical Care Corporation), Family Medicine - Tainan - Taiwan

**Keywords:** Snoring, Obstructive Sleep Apnea (OSA), Polysomnography (PSG)

## Abstract

**Objective:**

Snoring is a dominant clinical symptom in patients with obstructive sleep apnea (OSA), and analyzing snoring sounds might be a potential alternative to polysomnography (PSG) for the assessment of OSA. This study aimed to systematically examine the correlation between the snoring sounds and the apnea-hypopnea index (AHI) as the measures of OSA severity.

**Material and Methods:**

A comprehensive literature review using the MEDLINE, Embase, Cochrane Library, Scopus, and PubMed databases identified the published studies reporting the correlations between and severity of snoring and the AHI values by meta-regression analysis.

**Results:**

In total, 13 studies involving 3,153 adult patients were included in this study. The pooled correlation coefficient for snoring sounds and AHI values was 0.71 (95%CI: 0.49, 0.85) from the random-effects meta-analysis with the Knapp and Hartung adjustment. The *I*^2^ and chi-square *Q* test demonstrated significant heterogeneity (97.6% and *p*<0.001). After adjusting for the effects of the other covariates, the mean value of the Fisher’s *r*-to-*z* transformed correlation coefficient would have 0.80 less by the snoring rate (95%CI = -1.02, -0.57), 1.46 less by the snoring index (95%CI = -1.85, -1.07), and 0.21 less in the mean body mass index (95%CI = -0.31, -0.11), but 0.15 more in the mean age (95%CI = 0.10, 0.20). It fitted the data very well (*R*^2^=0.9641).

**Conclusion:**

A high correlation between the severity of snoring and the AHI was found in the studies with PSG. As compared to the snoring rate and the snoring index, the snoring intensity, the snoring frequency, and the snoring time interval index were more sensitive measures for the severity of snoring.

## INTRODUCTION

Snoring is a prevalent condition that greatly affects public health^1^. In the general population, the prevalence of chronic snoring is higher in men (40%) than in women (20%)^2^. Although not all people who snore have clinically significant obstructive sleep apnea (OSA), snoring is the earliest and most common symptom of OSA, occurring in 70% to 95% of patients with OSA^3^.

OSA is a serious sleep disorder that may cause deterioration in the quality of life, hypertension, and cardiovascular and cerebrovascular diseases^4^. A systematic review reported that the mean prevalence of OSA defined by an apnea hypopnea index (AHI) of ≥5 was 22% (9% to 37%) for men and 17% (4% to 50%) for women during 1993-2013^5^. However, OSA is undiagnosed in approximately 75% to 85% of persons with the condition^6^. Polysomnography (PSG) is regarded as the gold standard for OSA diagnosis and snoring monitoring^7^. PSG data can be used to measure OSA severity based on the AHI; severity is evaluated as follows: normal, AHI<5; mild, 5≤AHI<15; moderate, 15≤AHI<30; and severe, AHI≥30^8^.

Studies have increasingly drawn attention to analyzing the acoustic features of snoring sounds as a potential alternative to PSG in the diagnosis of OSA^9^. In recent years, researchers have attempted to develop a straightforward, economical test for diagnosing OSA through the analysis of snoring sounds^10^. The acoustic features of snoring sounds include intra-snore (snoring rate or duration, snoring index, snoring intensity, and snoring frequency) and inter-snore (Snore Time Interval Index, STII) features, and a combination of these. This study aimed to determine the correlation between snoring sounds and OSA severity according to the AHI.

## MATERIAL AND METHODS

The study protocol was approved by the Research Ethics Committee of the Buddhist Dalin Tzu Chi Hospital, Taiwan (No. B10703013). We followed the Preferred Reporting Items for Systematic Reviews and Meta-Analysis for Protocols 2015 (PRISMA-P 2015) guidelines to conduct this meta-analytic study^11^.

### Search strategy

We searched for English language articles using the MEDLINE, Embase, Cochrane Library, Scopus, and PubMed databases electronically from inception to October 20, 2020. We documented our literature search while conducting our systematic review. The first step of the procedure was formulating the subject of investigation, which was the correlation between snoring sounds and the AHI. The second step was formulating search terms according to patient, intervention, comparison, and outcome (PICO); the input for English synonyms was snor*.mp (mp = title, abstract, keyword), which included snore signal, snoring rate, snoring time, snoring duration, snoring frequency, snoring amplitude, and snoring intensity; or breath* ,np; furthermore, the outcome for English synonyms was the AHI, sleep apnea* .np, which included central sleep apnea, OSA, and sleep apnea syndrome. The third step was executing the searches in the aforementioned databases.

### Data collection and analysis

#### Selection of studies

Two authors, JKC and YHK, independently screened the titles, abstracts, and keywords of articles from the searches to identify potentially eligible papers. Any disagreements were resolved through consultation with CML.

#### Data collection

Data were first extracted into standardized forms by the 2 reviewers and subsequently extracted into a summary of findings tables. The reviewers were contacted to clarify any unclear data (methods of snoring detection) during data extraction. The methods of acoustic analyses of snoring during sleep are according to the acoustic characteristics of snoring, and classified as an intra-snore group, inter-snore group, and both intra- and inter-snore group. The severity of snoring was measured by the snoring intensity, snoring duration or snoring rate (snoring time/sleep time), and snoring index (or snoring burst index) for the intra-snore group and the snore time interval index (STII) for the inter-snore group in the collected studies. To examine the strength of the correlation between the severity of snoring and AHI, we recorded the estimated Pearson’s correlation coefficient from each of the collected studies as the measures of effect size.

#### Statistical analysis

Meta-analysis and meta-regression analysis were performed using the metaphor package of Viechtbauer in the R statistical software, version 4.0.3 (R Foundation for Statistical Computing, Vienna, Austria). A two-sided *p*-value≤0.05 was considered statistically significant.

As discussed by Borenstein et al. (2009)^12^, when the Pearson’s correlation coefficient, *r*, between two (normally-distributed) continuous variables was the measures of effect size, we did not perform meta-analysis on *r*’s because the variance of *r* depended strongly on *r* itself. Thus, we did the following instead: (1) first, each Pearson’s correlation coefficient, *r*, was converted to the Fisher’s *z* scale, (2) then, the meta-analysis was performed using the transformed *z* values, (3) finally, the results of meta-analysis, such as the summary effect and its 95% confidence interval (CI), were converted back to correlations for interpretation.

The random-effects meta-analysis of the Fisher’s *r*-to-*z* transformed correlation coefficient with the Knapp and Hartung adjustment was conducted to calculate the weighted average of individual-study correlations between snoring and AHI as the pooled summary effect to be illustrated in the forest plot. The heterogeneity across the collected studies was determined by the chi-square *Q* test and the *I*^2^ statistic, where the *p*-value of the *Q* test ≤0.15 or *I*^2^≥50% indicated a substantial amount of heterogeneity. If the statistical test of heterogeneity and the *I*^2^ statistic revealed substantial heterogeneity among the collected studies, a fixed-effects linear meta-regression model for modelling Fisher’s *r*-to-*z* transformed correlation coefficient was fitted to the meta-data by the weighted least squares (WLS) method to identify the relevant covariates (called the “moderators” in meta-analysis), which accounted for the observed heterogeneity. Moreover, if the statistical test for residual heterogeneity still revealed substantial heterogeneity among the collected studies, a mixed-effects linear meta-regression model of Fisher’s *r*-to-*z* transformed correlation coefficient was performed with the added random-effects to account for the unknown sources of heterogeneity.

To ensure the quality of analysis result, basic model-fitting techniques for (1) variable selection, (2) goodness-of-fit (GOF) assessment, and (3) regression diagnostics were used in our meta-regression analysis of Fisher’s *r*-to-*z* transformed correlation coefficient. Specifically, with the aid of the likelihood ratio test, the stepwise variable selection procedure (including iterations between the forward and backward steps) was applied to obtain the candidate final linear meta-regression^13^. Subgroup analyses for the different measures of the severity of snoring were performed to explore the heterogeneity. The reported coefficient of determination, *R*^2^, was calculated by computing the squared correlation between the observed and predicted Fisher’s *r*-to-*z* transformed correlation coefficient to assess the GOF of the fitted linear meta-regression model. Finally, the statistical tools of regression diagnostics for examination of publication bias, residual analysis, detection of influential studies, and check of multicollinearity were applied to discover any model or data problems. In particular, Egger’s test was used to examine the symmetry of the funnel plot for detecting publication bias.

## RESULTS

**Figure 1:**
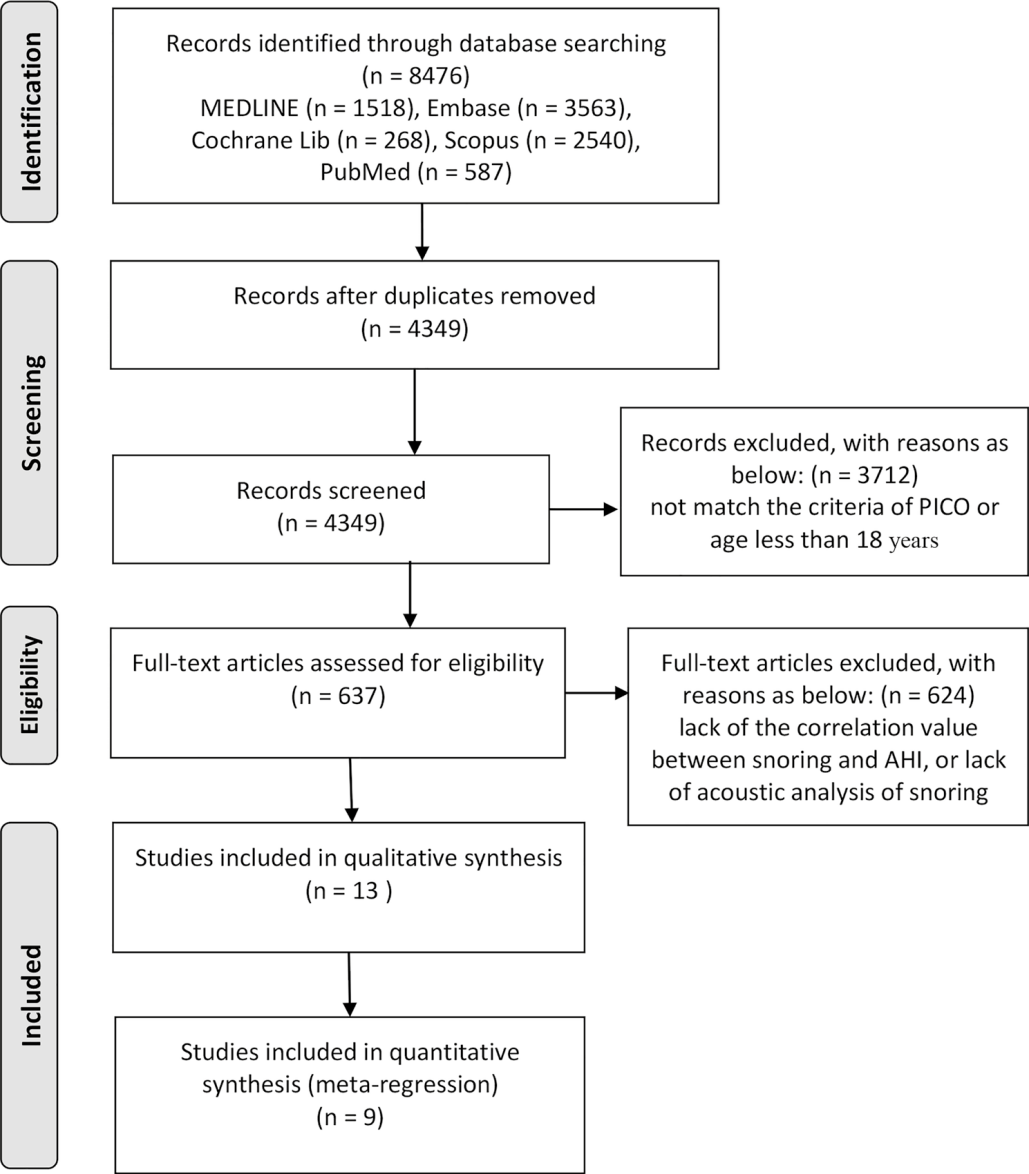
Flowchart of the study selection.

The searches yielded 1,518 papers in MEDLINE, 3,563 papers in Embase, 268 papers in the Cochrane Library, 2,540 papers in Scopus, and 587 papers in PubMed. The database from PubMed and OVID MEDLINE are the same and from MEDLINE, but the difference lied in the update speed and operating interface. Because PubMed was authoritative for many readers, we included these two databases to cite. All searches were conducted independently by 2 authors. A total of 8476 studies were screened for relevance; 4,127 studies were excluded because they were duplicates, 3,712 studies were excluded because they did not match the PICO criteria or because participants were younger than 18 years, 624 full-text articles were excluded due to a lack of correlation between snoring sounds and the AHI or a lack of acoustic snoring analysis. Figure 1 summarizes the flow chart for study selection. Finally, 13 studies involving 3,153 adult patients, the median number of participants (interquartile range) was 116 (90 to 211) and 5 out of 13 studies (38%) were prospective in design, were included for meta-analysis.

**Figure 2:**
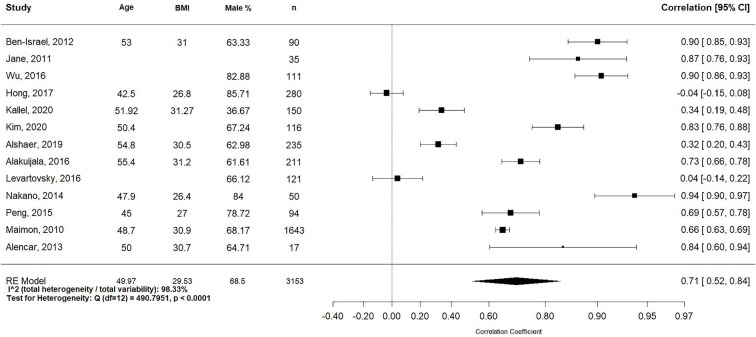
Forest plot for correlation between acoustic analysis of snoring and obstructive sleep apnea (OSA) in the thirteen studies selected.

The 13 studies provided data on 3,153 adult participants (weighted mean age, 49.97 years, weighted proportion of men, 68.5%, and weighted mean BMI 29.53kg/m^2^) in Figure 2, and details of these 13 studies are presented in Table 1. According to acoustic analysis methods, we classified these 13 studies into 5 subgroups. The first subgroup contained studies that analyzed snoring rate (or snoring duration); 3 studies reported correlations between snoring rates (snoring duration divided by total sleep time) and the AHI^14-16^. In these 3 studies, snore sensors were used to record snoring sounds during sleep. The corresponding researchers attempted to identify snoring sounds to estimate the snoring rate and then compared the estimated snoring rate to the AHI data obtained from PSG or other forms of sonography. The second subgroup contained studies that used a snoring index (snore number/sleep hour); 4 studies reported the correlations between AHI values and snoring index^17-20^. Snoring signals were estimated by recording the number of snores, which were detected by using either PSG or a special device at home. The third subgroup contained studies that analyzed snoring intensity; 4 studies reported correlations between snoring intensity and the AHI^3,10,17,21^. Snoring intensity was measured according to sound pressure level, peak dB level, or sound power dip, and then these estimated parameters were compared to AHI values obtained from PSG recordings. The fourth subgroup contained studies that analyzed snoring frequency; 3 studies reported correlations between the AHI and snoring sounds calculated according to snoring frequency^15,22,23^. The recorded sounds were segmented into numerous short windows, and the Mel frequency cepstral coefficient method, which has been widely applied in language recognition, was used to determine the snoring frequency. The fifth subgroup contained studies that used the STII. Two studies reported correlations between the AHI and the STII^22,24^. The STII method detected the number of inter-snoring episodes within a restricted duration (10s < snore time interval < 100s) as the possible apnea and then compared the STII with the AHI using PSG^24^. In Ben-Israel’s study^22^, snoring frequency and snoring index were applied to identify snoring, and in Jané’s study^17^, snore intensity, snoring frequency, and snoring index were used to identify snoring sounds. Characteristics of studies used for this systemic review of the correlation between the severity of snoring sounds and AHI were shown in Table 2.

**Table 1:** Characteristics of studies used for a systematic review of the correlation between the severity of snoring sounds and AHI.

Publication	Setting (Country)	Design	N, r	Snoring sounds measured according to acoustic features	AHI from PSG
Alakuijala and Salmi (2016)^16^	Finland	Cross-sectional study	N=211, r=0.727	The amount and percentage of snoring episodes (100ms/per episode) versus the total time in bed was calculated. The snoring rate percentages were then compared to the AHI values from cardiorespiratory polygraphy recordings.	AHI score was obtained from cardiorespiratory polygraphy according to AASM criteria.
Hong et al. (2017)^14^	South Korea	Retrospective case-control study	N=280, r=-0.038	Snoring rate was defined as the percentage of snoring time (from vibrating sensors) compared to total sleep time. These snoring rates were then compared to the AHI values from PSG recordings.	Yes
Kallel et al. (2020)^15^	Tunisia	Retrospective study	N=150, r=0.341	Snoring rate was defined as the percentage of snoring time compared to total sleep time. These snoring rates were then compared to the AHI values from respiratory polygraphy recordings.	AHI score was obtained from respiratory polygraphy according to AASM criteria.
Jané et al. (2011)^17^	Spain	Prospective study	N=35, r=0.87	This device (Snoryzer-Uno, S1) detects and automatically analyzes snoring intensity and frequency parameters to assess whether the acoustic characteristics of snoring sounds differ in patients with and without sleep apnea-hypopnea syndrome. The apnea index (AI) was obtained using S1, and then the estimated AIs were compared to the AHI values from PSG recordings.	Yes
Levartovsky et al. (2016)^20^	Israel	Cross-sectional study	N=121, r=0.04	Snoring index (SI) was defined as snoring events (intensity >50dB) per sleep hour. These SIs were then compared to the AHI values from PSG recordings.	Yes
Wu et al. (2016)^18^	Taiwan	Prospective study	N=111, r=0.905	The number of snoring burst signals was counted and then divided by the total sleep time to obtain the snoring burst index (SBI). The SBIs were then compared to AHI values from PSG recordings.	Yes
Alshaer et al. (2019)^19^	Canada	Cross-sectional study	N=235, r=0.32	The total number of snores per hour of sleep was considered as the SI. The SIs were then compared to the AHI values from PSG recordings.	Yes
Maimon e Hanly (2010)^3^	Canada	Prospective study	N=1643, r=0.66	The maximum decibel level recorded on the sound meter during each 30-s epoch of the polysomnogram test was identified, and the mean value of this measurement (mean maximum decibel level) during various sleep states and body positions was used to determine snoring intensity. These snoring intensities were then compared to the AHI values from PSG recordings.	Yes
Nakano et al. (2014)^21^	Japan	Cross-sectional study	N=50, r=0.94	Snoring intensity was assessed according to the highest one percentile ambient sound pressure level (L1) determined by a smartphone. The sound power dip was defined as a dip of more than a given threshold value in the time series, lasting ≤90s, with the descending and ascending portions steeper than the threshold value per 18s. The smartphone respiratory disturbance index (smart-RDI) was calculated as the number of smart-RDI values per hour. The smart-RDIs were then compared to the AHI values from PSG recordings.	Yes
Peng et al. (2015)^10^	China	Cross-sectional study	N=94, r=0.691	The average equivalent energy level of A-weighted sound over the test period (LAeq; dB), a measure of snoring intensity, was taken as a parameter. These LAeq values were then compared to the AHI values from PSG recordings.	Yes
Ben-Israel et al. (2012)^22^	Israel	Prospective study	N=90, r=0.9	Acoustic analysis based on intra- and inter-snore properties was performed using snoring frequency, SI, and STII to estimate the AHI values. These estimated AHI values were then compared with the AHI values from PSG recordings.	Yes
Kim et al. (2020)^23^	South Korea	Prospective cohort study	N=116, r=0.83	The features of sounds were extracted by a software (by random forest method) program based on snoring frequency, and the AHI was estimated. Then, these estimated AHIs were compared with those from PSG recordings.	Yes
Alencar et al. (2013)^24^	Brazil	Cross-sectional study	N=17, r=0.84	The STII was defined as Nδt/T (Nδt = number of snore time intervals for which 10s<δt<100s; T is the number of sleep hours). These STIIs were then compared to the AHI values from PSG recordings.	Yes

Abbreviation: AASM = American Academy of Sleep Medicine; AHI = Apnea hypopnea index; PSG = Polysomnography; STII = Snoring time interval index.

**Table 2:** Characteristics of studies used for a systematic review of the correlation between the severity of snoring sounds and AHI.

Publication	Male/Female	Age	BMI
Alakuijala and Salmi (2016)^16^	130/81	55.4±14.0	31.2±6.5
Hong et al. (2017)^14^	240/40	42.5±0.8	26.8±0.2
Kallel et al. (2020)^15^	55/85	51.9±10.6	31.3±5.7
Jané et al. (2011)^17^	35	-	-
Levartovsky et al. (2016)^20^	80/41	-	-
Wu et al. (2016)^18^	92/19	-	-
Alshaer et al. (2019)^19^	148/87	54.8±15.0	30.5±7.1
Maimon e Hanly (2010)^3^	1120/523	48.7±13.7	30.9±8.8
Nakano et al. (2014)^21^	42/8	47.9±13.7	26.4±6.1
Peng et al. (2015)^10^	74/20	45	27
Ben-Israel et al. (2012)^22^	57/33	53±13.0	31±5.0
Kim et al. (2020)^23^	78/38	50.4±16.7	-
Alencar et al. (2013)^24^	11/6	50±10.0	30.7±6.7
Summary statistic	68.73%	49.96±4.11	29.53±2.12

**Figure 3:**
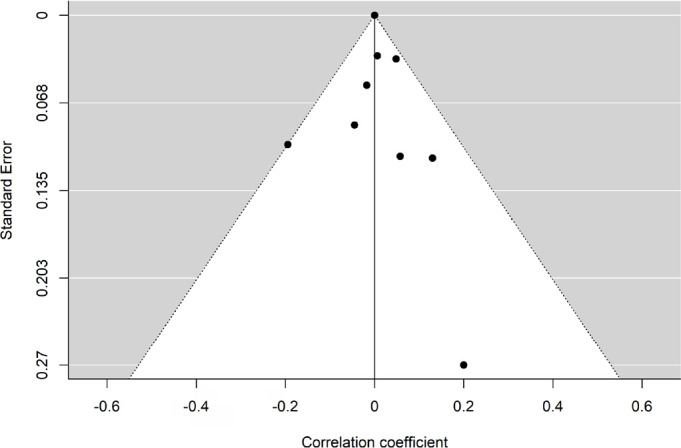
Funnel plot for the assessment of potential publication bias after adjustments by meta-regression analysis.

Both the *I*^2^ statistic (total heterogeneity/total variability; 97.6%) and Cochran’s Q test (*p*<0.001) demonstrated significant heterogeneity for all 13 studies. The pooled correlation coefficient of the random effects model for snoring sounds and AHI was 0.71 (95%CI = 0.49, 0.85, *p*<0.001; Figure 2). Figure 3 displays the funnel plot. The Egger’s test failed to reject the null hypothesis of asymmetry in the funnel plot (*p*=0.55), indicating that there was no strong enough evidence for the presence of publication bias.

The influential analysis omitted Hong’s study^14^, and this resulted in a pooled effect of 0.96 (95%CI = 0.72, 1.20). For the leave-one-out analysis, the mean pooled effect was 0.89 ± 0.05 (range: 0.82, 0.96). We further conducted subgroup analyses according to snoring features. The pooled effects of correlations between the AHI and the various subgroups according to the snoring rate, the snoring index, the snoring intensity, the snoring frequency, and the STII were 0.39 (95%CI = -0.17, 0.76, *p*=0.168), 0.66 (95%CI = 0.11, 0.90, *p*=0.023), 0.82 (95%CI = 0.64, 0.91, *p*=0.014), 0.87 (95%CI = 0.83, 0.89, *p*<0.001), and 0.89 (95%CI = 0.85, 0.93, *p*<0.001), respectively. Finally, as shown in Table 3, we performed a mixed-effects linear meta-regression analysis of the Fisher’s *r*-to-*z* transformed correlation coefficient from the 9 studies without missing values to identify the predictors for the correlation between the severity of snoring and AHI. After adjusting for the effects of the other covariates, the mean value of the Fisher’s *r*-to-*z* transformed correlation coefficient would have 0.7951 less in the studies with the severity of snoring measured by the snoring rate (95%CI = -1.02, -0.57, *p*<0.001), 1.4595 less in the studies with the severity of snoring measured by the snoring index (95%CI = -1.85, -1.07, *p*<0.001), and 0.2094 less per unit of increment in the study-level mean body mass index (95%CI = -0.31, -0.11, *p*=0.004), but 0.1510 more per year of increment in the study-level mean age (95%CI = 0.10, 0.20, *p*<0.001). Thus, as compared to the snoring rate and the snoring index, the snoring intensity, the snoring frequency, and the STII were more sensitive measures for the severity of snoring. This multiple mixed-effects meta-regression model was fitted using the restricted, residual, or reduced maximum likelihood (REML) method with the Knapp and Hartung adjustment (*k*=9). The Knapp and Hartung adjustment were specifically used in fitting mixed-effects meta-regression models with the number of observations *k*<30. The test for residual heterogeneity, *χ*^2^ statistic (df=4) = 6.01, *p*=0.198>0.15. And, *R*^2^=0.964 indicated an excellent fit.

**Table 3:** Multivariable meta-analysis of the predictors for the correlations between severity of snoring and the apnea-hypopnea index (AHI) by fitting a mixed-effects linear meta-regression model of Fisher’s r-to-z transformed correlation coefficient to 9 studies with the stepwise variable selection method (2,770 participants)^*^.

Covariate	Estimate	Standard error	t-value	p-value	95% confidence interval
Intercept	-0.0964	0.5977	-0.1613	0.8797	-1.7557, 1.5603
Snoring rate	-0.7951	0.0825	-9.6398	0.0006	-1.0241, -0.5661
Snoring index	-1.4595	0.1397	-10.4487	0.0005	-1.8474, -1.0717
Mean age (years)	0.1510	0.0172	8.7931	0.0009	0.1033, 0.1986
Mean body mass index	-0.2094	0.0362	-5.7844	0.0044	-0.3099, -0.1089

Notes: ^*^Mixed-effects meta-regression model with the Knapp and Hartung adjustment (*k* = 9; *τ*^2^ estimator: ML), test for residual heterogeneity, χ^2^ statistic (df=4) = 6.0120, *p*-value=0.1983>0.15, where ML stands for the maximum likelihood estimation method. And, *R*^2^ = 0.9641 indicated an excellent fit. This analysis was performed using the escalc() and rma() functions of the metafor package in R (version 4.0.4).

## DISCUSSION

In our analysis, we revealed a high correlation between snoring sounds and OSA severity according to the total AHI (pooled correlation: 0.71, 95%CI = 0.49 to 0.85) in a group with performing PSG test. For this systematic review, an expansive search of the literature was conducted, and 13 studies were identified that reported correlations between snoring sounds and the AHI in the studies with PSG. We also found that less by the snoring rate, less by the snoring index and less per unit of increment in the study-level mean BMI, but more per year of increment in the study-level mean age were the significant factors for the correlation between snoring and AHI among our collected articles by meta-regression analysis. To the best of our knowledge, this is the first meta-analysis to synthesize the results of the correlations between snoring and AHI from empirical studies.

There were many methods including questionnaires and objective measures those helped determine the probability that a patient had OSA. Patients with snoring and suspicion of OSA were suggested to perform a PSG test to diagnose OSA. PSG remained the gold standard for OSA diagnosis. Snoring is highly correlated with OSA in current study. We recommended patient with snoring needed to visit clinician to performed PSG to confirm the diagnosis of OSA. Since PSG is highly time-consuming, expensive, and only available in the medical facilities, if the correlation between the snoring and the AHI is high, we may use the measures of snoring to assess the OSA severity. Furthermore, patients received some treatments for OSA, how to follow up the treatment effects frequently and truly at home was our concern. Frequently repeated testing by PSG was inconvenient for most of patients. For home testing, digital recording and analyzing the sound signals was an alternative method to PSG.

Many researchers have attempted to develop a straightforward, economical test for diagnosing OSA by analyzing snoring sounds^10^. One study reported that snoring is a positive predictor of OSA^25^. A systematic review reported that the clinical symptoms of nocturnal gasping and choking are the most reliable indicators of OSA, whereas snoring was not particularly indicative^26^. In this study, snoring sounds were recorded using various methods to analyze the acoustic features of snoring. We revealed a high correlation between snoring sounds and OSA severity according to the AHI. Therefore, patients with OSA could record their snoring behavior at home as an alternative approach to follow-up treatment because PSG is not convenient for follow-up. Additionally, we found that STII, snoring frequency, and snoring intensity might be an appropriate alternative approach for measuring AHI values.

We further analyzed the correlation between the AHI and snoring sounds according to 5 subgroups and found that STII (*r*=0.89) resulted in the highest correlation, followed by snoring frequency (*r*=0.87). Measuring AHI using a snoring rate resulted in the lowest correlation (*r*=0.39). Although we revealed that employing snoring rate and snoring index resulted in correlations with AHI values determined from PSG, the estimate of correlations was less than others (STII, snoring frequency, and snoring intensity). This may be due to the deviation between the correlations, such as the negative r (-0.04) in Hong’s study^14^ as well as the lower correlations (0.04) in Levartovsky’s study^20^ and Alshaer’s study^19^ (0.32). Another explanation might be the limited number of studies.

We further investigated the significant factors for the correlation of the snoring and AHI by fitting a multiple linear meta-regression model. We found that the mean age of participants was ranged from 42.5 to 55.4 years, and age was positive association with the correlation between snoring and AHI. For adults, overweight was defined as a BMI 25.0 or higher, and obesity as a BMI 30.0 or higher^27^. We also found that BMI was range from 26.4 to 31.3, and BMI was a negative association with the correlation between snoring and AHI. The possible explanation might be the different methods of snoring detection.

The prevalence of chronic snoring is higher in men than women^2^, and obstructive sleep apnea is more common in men than in women^28^. Twelve studies of our current meta-analysis showed the correlations of snoring and AHI based on both genders. Only one study reported the correlation of snoring percentage and AHI was slightly stronger among women (*n*=81, *r*=0.78) than men (*n*=130, *r*=0.69)^16^. However, no reports of these 13 studies showed the prevalence of snoring for each gender respectively.

The percentage of individuals with primary snoring sounds was 15.1% (475/3,153) in the current study. Our pooled correlation would be stronger if this percentage (15.1%) could be deleted. Further research should investigate the features between primary snoring and OSA snoring.

There are some limitations in current study. First, although 5 articles mentioned small sample size as a limitation and differing trends between snoring and AHI, this meta-analysis (total participants = 3,153) revealed a high correlation between snoring and the AHI^15,18-20,24^. Second, the participants of certain studies were referred by individuals in different clinical settings to visit sleep centers and undergo PSG to diagnose OSA. Therefore, 85.3% individuals with snoring sounds had OSA in this study. Third, we further investigated the significant factors for correlation between the snoring and AHI by fitting a meta-regression model. However, the mean age and BMI were not available in four articles. Finally, 11 studies used standard AHI values from PSG, and 2 studies used AHIs from respiratory and cardiorespiratory polygraphy^15,16^. However, these 2 polygraphy recordings were scored according to criteria established by the American Academy of Sleep Medicine^29^.

In conclusion, we revealed a high correlation between snoring sounds and OSA severity according to total AHI in a group with performing PSG test. The various methods used to analyze snoring sounds included snoring rate, snoring index, snoring intensity, snoring frequency, and STII. We discovered that snoring frequency and STII had the highest correlations. We also found that less by the snoring rate, less-by the snoring index and less mean BMI, but more per year of increment in mean age were the significant factors for the correlation between snoring and AHI among our collected articles by meta-regression analysis.

## Abbreviation

AASM: American Academy of Sleep Medicine

AHI: apnea hypopnea index

BMI: body mass index

95%CI: 95% confidence interval

GOF: goodness-of-fit

OSA: sleep apnea syndrome

PICO: patient, intervention, comparison, and outcome

PSG: Polysomnography

STII: the snore time interval index.
